# Joint Optimization of Trajectory-Resource Allocation and Deep Task Partial Offloading for MEC-Enabled Multi-UAV

**DOI:** 10.3390/s26113540

**Published:** 2026-06-03

**Authors:** Chuanjie Liu, Yangjun Wang, Haibo Mei, Shuang Du, Bing Guo

**Affiliations:** 1 School of Computer Science, Sichuan University, Chengdu 610065, China; 2021323045045@stu.scu.edu.cn; 2 School of Aeronautics and Astronautics, University of Electronic Science and Technology of China, Chengdu 611731, China; 202421100410@std.uestc.edu.cn (Y.W.); haibo.mei@uestc.edu.cn (H.M.); sdu@uestc.edu.cn (S.D.)

**Keywords:** UAV communications, mobile edge computing (MEC), joint optimization, UAV trajectory, unmanned aerial vehicle (UAV), Partial Program Offloading (PPO)

## Abstract

Currently, multiple unmanned aerial vehicles (UAVs) can cooperatively work as mobile edge computing (MEC) servers in the sky to provide computation services to ground terminals (GTs). Such an MEC-enabled multi-UAV system will greatly benefit the GTs, each of which can offload its tasks on demand to a nearby UAV. In particular, if a GT has to process computation-intensive deep learning tasks in a catastrophic environment, it can partially offload these tasks to UAVs using a scheme like Partial Program Offloading (PPO). This ensures the quick processing of the deep learning tasks while saving computing resources on both the GT and UAV sides. Nevertheless, UAV–GT offloading links are frequently blocked by ground obstacles in complicated environments, and individual UAVs may have limited computation capacity. Moreover, UAVs lack a constant propulsion energy supply to sustain a long mission time. All these factors lead to a degraded Quality of Service (QoS) for GTs in terms of task latency. To address this issue, we propose to jointly optimize the UAV trajectories, computing resource allocation, and the partial offloading of deep learning tasks. The formulated joint optimization problem is challenging to solve optimally, as it is non-convex and involves multiple coupled constraints. We propose utilizing the Successive Convex Approximation (SCA) method alongside a Block Coordinate Descent (BCD) approach to tackle this joint problem. Numerical results demonstrate that the proposed joint optimization scheme significantly outperforms the benchmark solutions.

## 1. Introduction

Unmanned aerial vehicles (UAVs) have been increasingly deployed in diverse domains, including surveillance, disaster response, and wireless communications [1,2]. In the context of wireless networks, UAVs offer unique advantages over fixed terrestrial infrastructure: they can be rapidly deployed in three-dimensional space, dynamically adjust their positions to establish favorable line-of-sight (LoS) links with ground terminals (GTs), and serve as mobile edge computing (MEC) servers to process computation-intensive tasks on behalf of resource-constrained GTs. This MEC-enabled multi-UAV paradigm is particularly valuable in time-critical scenarios—such as post-disaster search and rescue—where GTs must execute deep learning tasks (e.g., real-time object recognition for locating trapped personnel) under stringent latency constraints but lack sufficient local computing power.

However, realizing efficient MEC-enabled multi-UAV systems requires addressing several tightly coupled challenges that existing works have not jointly resolved. First, UAV–GT communication links in urban or cluttered environments are frequently obstructed by ground obstacles, causing significant path loss variations that depend on the UAV’s instantaneous position relative to each GT [3,4]. Second, UAV propulsion energy is strictly limited, and the flight trajectory directly determines both the communication link quality and the energy budget available for computation services [5]. Third, deep learning tasks possess a unique sequential layer-wise structure: unlike general computation tasks that can be treated as indivisible units, deep neural networks (DNNs) can be partitioned at intermediate layers, enabling partial offloading where the front layers execute locally and the remaining layers execute on the UAV. This Partial Program Offloading (PPO) introduces additional coupling between the offloading ratio, the required uplink data volume (including intermediate results), and the UAV’s computing resource allocation. These interdependencies—trajectory affecting link quality, link quality constraining offloading feasibility, offloading ratio determining computing demands, and computing demands influencing energy consumption—form a complex joint optimization problem that cannot be decomposed into independent subproblems.

Existing research has made progress in individual dimensions of this problem but falls short of addressing the full coupling. In UAV trajectory optimization, prior works have focused on maximizing communication throughput or minimizing propulsion energy without considering MEC task demands [5,6,7,8]. In MEC-enabled UAV systems, studies have explored task offloading and multi-UAV collaboration [9,10,11], but most adopt binary (full) offloading and single-dimension optimization. Recent Deep Reinforcement Learning (DRL) approaches [12,13] have attempted joint optimization but require extensive training (over 10,000 episodes) and lack convergence guarantees, making them unsuitable for time-sensitive missions. Critically, no existing work jointly optimizes UAV trajectories, computing resource allocation, and *partial* deep learning task offloading in a multi-UAV setting—the specific gap this paper addresses.

To fill this gap, we formulate a joint optimization problem that minimizes the maximum energy consumption across all UAVs while satisfying the QoS requirements (task completion deadlines) of all GT deep learning tasks. The optimization jointly determines (i) the flight trajectory of each UAV, (ii) the GT-UAV association for task offloading, and (iii) the computing resource allocation at each UAV. The resulting problem is non-convex with multiple coupled constraints. We propose a block coordinate descent (BCD) algorithm combined with successive convex approximation (SCA) to decompose and iteratively solve this problem. The BCD framework is chosen over DRL-based alternatives for three reasons: it provides deterministic convergence guarantees to a stationary point, achieves polynomial-time complexity per iteration without requiring offline training, and converges within approximately 50 iterations (3–5 s wall-clock time), ensuring real-time applicability for time-sensitive UAV missions.

The main contributions of this paper are summarized as follows:

1. We design a Partial Program Offloading (PPO) scheme tailored for deep learning tasks in MEC-enabled multi-UAV systems. Unlike binary offloading, PPO splits DNN tasks at an optimal layer, enabling flexible workload distribution between GTs and UAVs. We formulate a joint optimization problem that couples UAV trajectories, GT–UAV associations, computing resource allocation, and partial offloading ratios under energy and latency constraints.
2. We develop an iterative SCA-BCD algorithm to solve the formulated non-convex problem. The algorithm decomposes the joint problem into three tractable subproblems—GT-UAV association, computing resource allocation, and trajectory optimization—each solved via convex programming in each iteration. We prove that the algorithm converges monotonically to a suboptimal solution within a prescribed accuracy.
3. We validate the proposed algorithm through extensive simulations. The results demonstrate that the joint optimization significantly reduces UAV energy consumption compared to baseline (elliptical trajectory) and TSP-based solutions, while satisfying all GT task latency deadlines. We further show the necessity of partial offloading by demonstrating that binary offloading becomes infeasible under the same resource constraints.

The remainder of this paper is organized as follows. Section 2 reviews related work on UAV trajectory optimization, MEC task offloading, and DRL-based approaches. Section 3 presents the system model and problem formulation. Section 4 details the proposed SCA-BCD solution. Section 5 provides simulation results and analysis. Section 6 concludes the paper and discusses future directions.

## 2. Related Work

### 2.1. UAV Trajectory Optimization

UAV trajectory design is fundamental to UAV-assisted communication systems, as the flight path directly determines link quality, coverage, and energy efficiency. Early research primarily addressed static or quasi-static UAV placement in three-dimensional space to maximize coverage or minimize path loss [6,7,8,14,15]. These works demonstrated that careful positioning of UAVs can significantly enhance ground user connectivity, yet they largely treated the UAV as a stationary relay without exploiting its mobility.

Subsequent studies shifted toward dynamic trajectory optimization to improve communication performance. Joint optimization of UAV motion control and user scheduling has been explored to maximize aggregate data rates [16], while other works revealed that UAV flight perturbations degrade link stability and proposed height-trajectory co-optimization to improve average channel capacity [17]. Energy-efficient trajectory design has also attracted considerable attention: Dai et al. [5] developed a generalized propulsion energy model for UAV communications, and resource-efficient multi-hop UAV path planning has been studied to maintain seamless connectivity during transitions [18]. These trajectory-centric works, however, primarily optimize communication metrics (throughput, coverage, or propulsion energy) in isolation, without jointly considering the computational demands imposed by MEC task offloading—particularly for structured deep learning workloads that require layer-wise partitioning.

The integration of UAVs with 5G/6G cellular infrastructure has further expanded the design space. Comprehensive surveys [3,4] have outlined the roadmap from current UAV-cellular integration toward future autonomous aerial networks, emphasizing the need for joint communication-computation-trajectory co-design. Buffer-aided relay strategies [19] and distributed-learning-based swarm coordination [20] represent additional directions, yet none of these works address the unique coupling between trajectory planning and partial offloading of computation-intensive deep learning tasks.

### 2.2. MEC-Enabled UAV Task Offloading

Mobile edge computing extends UAV capabilities beyond communication relaying to on-board task processing. Several studies have explored UAV-mounted MEC servers that accept offloaded tasks from ground terminals [9,10]. Aerial collaboration platforms leveraging distributed Q-learning have been proposed to coordinate task allocation among UAV swarms for improved energy efficiency [11]. Federated learning frameworks further enable privacy-preserving distributed intelligence across multi-UAV systems for applications such as collaborative sensing and environmental monitoring [21,22]. Computing power networking concepts [23] have also been introduced to orchestrate heterogeneous computing resources across UAV fleets.

Despite these advances, a critical gap persists in handling *deep learning task offloading* as opposed to general computation offloading. Deep learning tasks exhibit unique characteristics that distinguish them from conventional MEC workloads: they possess a sequential layer-wise execution structure where intermediate results must be transmitted between partition points; they demand heterogeneous computing resources across different layers, and their data volumes vary significantly depending on the chosen split point within the neural network architecture. These properties make binary (all-or-nothing) offloading strategies inefficient, as full offloading may overwhelm limited UAV bandwidth and computing capacity, while purely local execution fails to meet stringent latency deadlines. Partial Program Offloading—where the deep learning model is split at an optimal layer and the remaining layers are executed remotely—offers a promising middle ground, yet its integration with multi-UAV trajectory and resource optimization remains largely unexplored.

### 2.3. Deep Reinforcement Learning Approaches

Deep Reinforcement Learning (DRL) has emerged as a popular paradigm for UAV communication and MEC optimization. DDPG-based methods have been applied to maximize UAV service time and throughput [24], while Double Deep Q-Network (DDQN) algorithms have been used to optimize trajectory and connection sequencing [25]. In the MEC domain, DRL-based secure communication [9] and energy-efficient edge computing with heterogeneous mixture-of-experts architectures [26] represent recent advances. Li et al. [12] proposed a triple-learner reinforcement learning approach for joint trajectory planning, application placement, and energy renewal in UAV-MEC systems, while their subsequent work [13] integrated RL with stochastic game theory for energy-efficient UAV swarm scheduling with dynamic clustering.

However, DRL-based approaches face inherent limitations for time-sensitive MEC-UAV missions: they typically require thousands of training episodes to converge, exhibit sensitivity to hyperparameter tuning, and struggle to provide convergence guarantees. In contrast, optimization-based methods such as successive convex approximation (SCA) combined with block coordinate descent (BCD) offer deterministic convergence properties, polynomial-time complexity per iteration, and real-time adaptability without offline training. This motivates our choice of an SCA-BCD framework, which achieves convergence within approximately 50 iterations (3–5 s on standard hardware) while jointly optimizing trajectory, resource allocation, and partial deep learning task offloading—a triple-dimension coupling that existing DRL works have not addressed simultaneously.

To clearly illustrate the differences between this work and existing studies, we summarize the key features in Table 1.

## 3. System Model and Problem Formulation

**Figure 1:**
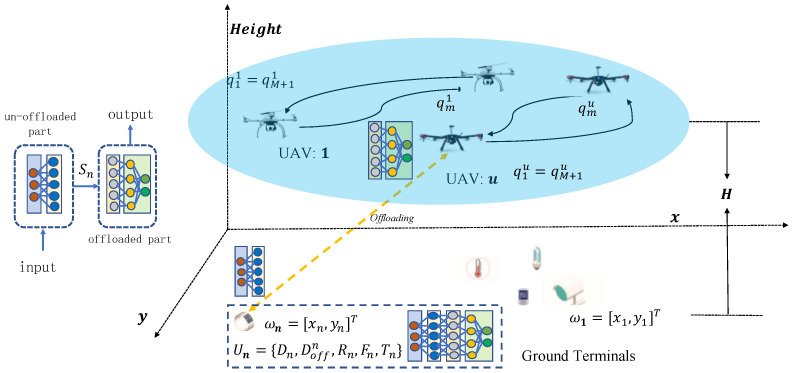
The architecture of MEC-enabled Multi-UAV system.

The architecture of MEC-enabled Multi-UAV system is shown in Figure 1, where U≜{1,2,…,U} rotary-wing UAVs are deployed to work as MEC servers to serve GTs in the field, and there are N≜{1,2,…,N} static GTs on the ground with $w_{n}={\left[x_{n},y_{n}\right]}^{T}\in R^{2\times 1}$, with ∀n being the horizontal location of the *n*-th GT. In this paper, we consider only rotary-wing UAVs, as they can hover statically in the air to establish stable UAV–GT links—a capability that fixed-wing UAVs lack. We assume that each UAV and GT is equipped with a single omni-directional antenna, and the UAV–GT link operates in half-duplex mode. For clarity, the key notation used throughout this paper is summarized in Table 2.

### 3.1. System Model

#### 3.1.1. UAV Trajectory and Flight Model

To model the UAV trajectory, we discretize the mission time into *M* equal time slots, each of duration $t_{s}$. The UAV path is thus represented by M+1 waypoints in 3D coordinates: ${\left\{H,q_{u}\left[m\right]\right\}}_{m=1}^{M+1}$. Here, m∈M≜{1,2,…,M+1} denotes the waypoint index (i.e., the *m*-th discrete time slot boundary), *H* is the fixed flight altitude common to all UAVs, and $q_{u}\left[m\right]=\left(x_{u}\left[m\right],y_{u}\left[m\right]\right)$ represents the horizontal coordinate of the *u*-th UAV at the *m*-th waypoint. We impose the return constraint $q_{u}\left[1\right]=q_{u}\left[M+1\right]$ to ensure each UAV returns to its initial position after the mission, which is the most common operational requirement. The fixed altitude *H* is chosen to guarantee obstacle-free straight-and-level flight while maintaining reasonable proximity to GTs.

The maximum horizontal displacement per segment is constrained as $∥q_{u}\left[m+1\right]-q_{u}\left[m\right]∥\leq \Delta_{\max}^{h},\;m=1,\ldots ,M$, where $\Delta_{\max}^{h}=t_{s}\cdot V_{\max}^{h}$ is determined by the product of the segment duration and the maximum horizontal velocity of the UAV. This value is set based on the physical speed limit of the rotary-wing UAV platform (typically 10–20 m/s for commercial multi-rotor UAVs [5]). With this constraint, the *u*-th UAV flies with approximately constant horizontal velocity within each segment, and the distance between the UAV and each GT remains approximately unchanged within each segment. The total number of segments *M* must be sufficiently large to satisfy $M\times \Delta_{\max}^{h}\geq \hat{D}$, where $\hat{D}$ is a lower bound of the required total UAV flying distance.

Let $t_{s}$ denotes the fixed duration that the UAV remains in each line segment. Then, the total mission completion time can be denoted as $\sum_{m=1}^{M}t_{s}$. The horizontal flying velocity of the *u*-th UAV along the *m*-th line segment is thus given by $v_{u}\left[m\right]=\frac{\sqrt{{∥q_{u}\left[m+1\right]-q_{u}\left[m\right]∥}^{2}}}{t_{s}}\leq V_{\max}^{h}$, m=1,…,M, where $V_{\max}^{h}$ is the maximum horizontal velocity of the UAV in *m*-th line segment. With the UAV velocity, for the *u*-th rotary-wing UAV, the propulsion energy cost in *m*-th line segment can be modeled as

(1) $$\begin{array}{cc} \; & E_{u}^{r-\mathrm{uav}}\left[m\right]=P_{0}\left(1+\frac{3{\left(v_{u}\left[m\right]\right)}^{2}}{U_{\mathrm{tip}}^{2}}\right)+\frac{1}{2}d_{0}\rho sG{\left(v_{u}\left[m\right]\right)}^{3}\\ \; & +P_{1}{\left(\sqrt{1+\frac{{\left(v_{u}\left[m\right]\right)}^{4}}{4\upsilon_{0}^{4}}}-\frac{{\left(v_{u}\left[m\right]\right)}^{2}}{2\upsilon_{0}^{2}}\right)}^{\frac{1}{2}} \end{array}$$

where $P_{0}$ and $P_{1}$ are constants representing blade profile power (related to rotor friction in hover) and induced power (related to lift generation in hover), respectively. $U_{\mathrm{tip}}$ denotes rotor blade tip speed, $v_{0}$ is the mean rotor-induced velocity in hover (linked to lift requirements), $d_{0}$ is the fuselage drag ratio (indicating streamline efficiency), *s* is rotor solidity (ratio of total blade area to rotor disc area), ρ is air density, and *G* is the rotor disc area. The UAV’s propulsion energy depends on its horizontal velocity in each segment. For simplicity, we ignore acceleration/deceleration energy consumption.

#### 3.1.2. UAV–GT Communication

As discussed before, a UAV–GT link may be blocked by ground obstacles. Thus, during the task offloading through uplink, we have to take the effect of the environment on the occurrence of LoS into consideration. Specifically, adopting an air-to-ground channel model in urban environments, the LoS probability of a UAV–GT link is given as

(2) $$\begin{array}{cc} \; & p_{un}\left[m\right]=\frac{1}{1+a\exp\left(-b\left(\arctan\left(\frac{H}{d_{un}\left[m\right]}\right)-a\right)\right)} \end{array}$$

where *a* and *b* are constant values that depend on the environment. In this setting, the altitude and antenna heights of the GT are neglected. Specifically, the probability of having LoS for GT *n* depends on the altitude of the UAV *H* and the horizontal distance between the UAV *u* and GT *n* at time slot *m* denoted as $d_{un}\left[m\right]=\sqrt{{∥q_{u}\left[m\right]-w_{n}∥}^{2}}$. Then the pathloss expression of the UAV–GT link becomes

(3) $$\begin{array}{cc} \; & l_{un}\left[m\right]=20\log\left(\sqrt{H^{2}+{\left(d_{un}\left[m\right]\right)}^{2}}\right)+Ap_{un}\left[m\right]+C \end{array}$$

where *A* and *C* are constants such that $A=\eta_{\mathrm{LoS}}-\eta_{\mathrm{NLos}}$ and $C=20\log\left(\frac{4\pi f_{c}}{c}\right)+\eta_{\mathrm{NLos}}$; $f_{c}$ is the carrier frequency (Hz); *c* is the speed of light (m/s); and $\eta_{\mathrm{LoS}}$ and $\eta_{\mathrm{NLoS}}$ (in dB) are, respectively, the losses corresponding to the LoS and non-LoS connections depending on the environment. Based on (3), the instantaneous achievable rate of the *n*-th GT’s uplinking to the *u*-th UAV in path line *m*, can be expressed in bits/second (bps) as

(4) $$\begin{array}{c} r_{un}\left[m\right]=B\log_{2}\left(1+\frac{P10^{\frac{-l_{un}\left[m\right]}{10}}}{BN_{0}}\right) \end{array}$$

where $N_{0}$ denotes the power spectral density of the Additive White Gaussian Noise (AWGN) at the receivers; *P* is the transmit power allocated by each GT; *B* denotes the total available system bandwidth in Hertz (Hz). It is worth noting that while the UAVs and GTs are equipped with omni-directional antennas, co-channel interference is not explicitly factored into Equation (4) (i.e., it utilizes an SNR rather than an SINR model). This assumption is predicated on the premise that the system employs Orthogonal Frequency-Division Multiple Access (OFDMA). Under OFDMA, the total available frequency band can be partitioned into multiple orthogonal sub-channels. By allocating non-overlapping sub-channels to adjacent UAVs and GTs, co-channel interference is effectively avoided. Theoretically, if dynamic sub-channel allocation were considered, the bandwidth parameter *B* in Equation (4) would be scaled by a fractional coefficient. However, to maintain focus on the core joint optimization of trajectory, computing resources, and partial offloading, we assume orthogonal sub-channels are pre-allocated and omit the explicit sub-channel allocation modeling in this paper.

Finally, we define the binary GT-UAV association variable $\alpha_{un}\in \left\{0,1\right\}$, where the subscript *u* indexes the UAV (u∈U) and *n* indexes the GT (n∈N). Specifically, $\alpha_{un}=1$ indicates that the *n*-th GT is associated with and offloads its deep learning task to the *u*-th UAV; $\alpha_{un}=0$ otherwise. Each GT can associate with at most one UAV during the entire mission, i.e.,

(5) $$\begin{array}{c} \sum_{u=1}^{U}\alpha_{un}⩽1,\forall n \end{array}$$

which indicates that one GT will associate and offload its deep learning task to one UAV during the whole mission time, as shown in Figure 1.

#### 3.1.3. Offload Deep Learning Task

We consider that each GT offloads a deep neural network (DNN) task with sequential execution structure to a UAV in this paper. As illustrated in Figure 1, all the layers of the DNN task will be executed in a strict order, and the execution of the latter layer requires the result of the previous layer as the input. Assume the intermediate results produced by the previous layer are non-trivial and cannot be ignored during offloading. Assume one *n*-th GT has an expected deep learning task $U_{n}=\left\{D_{n},D_{off}^{n},R_{n},F_{n},T_{n}\right\}$ during the whole mission time, where $D_{n}$ (in Mb) and $F_{n}$ (in GHz/Mb) represent the amount of data and the required computing resource of the task, respectively. $T_{n}$ denotes the task’s completion deadline. $R_{n}$ is the ratio of the size of the task-output data to that of the task-input data. Here, each deep learning task is divided into two parts with different sizes. The first part, with size $D_{n}-D_{off}^{n}$, is executed locally at the GT, while the second part, with size $D_{off}^{n}\in \left[0,D_{n}\right]$, is offloaded to an UAV for remote execution. Since the second part of the task can be executed only when the intermediate result produced by the first part is obtained, we use $S_{n}=D_{n}\times R_{n}$ to represent the amount of data of the intermediate result. In addition, assume the task offloading will not take place in the stages of UAV taking off and landing, which is in a relatively short period. Thus, the UAV trajectory in the stages of UAV taking off and landing will not be considered in the system model. We consider each GT offloads a deep neural network (DNN) task—typically common computation-intensive tasks in emergency, field, or remote-service scenarios, such as object recognition (e.g., locating trapped personnel in post-disaster areas via lightweight DNN models)—with a sequential execution structure to the UAV in this paper.

**Figure 2:**
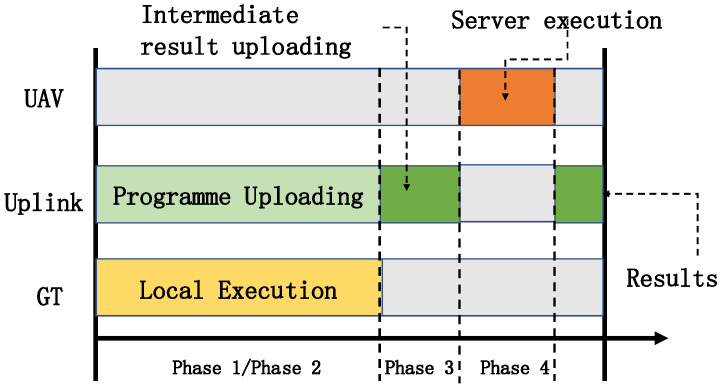
GT follows the Partial Program Offloading (PPO) Scheme to offload tasks to the UAV.

As shown in Figure 2, this paper follows the Partial Program Offloading (PPO) scheme, which divides the task offloading process from one GT to its associated UAV in four phases: local execution, program uploading, uploading of intermediate results, and UAV server execution. For phase one, local execution, let $f_{n}^{lc}$ in (GHz) denote the processing speed of the computing unit of the *n*-th GT. The time taken by the GT to complete the local part of the deep learning task in phase one is given by

(6) $$\begin{array}{c} T_{n}^{lc}=F_{n}\left(D_{n}-D_{off}^{n}\right)/f_{n}^{lc} \end{array}$$

According to the PPO scheme, within $T_{n}^{lc}$, the *n*-th GT has to strictly complete phase two, i.e., program uploading, to realize efficient parallel processing. It is worth noting that to avoid redundant transmission and minimize latency, the UAVs are assumed to have pre-cached the standard, static deep learning model backbones. Therefore, the offloaded data $D_{off}^{n}$ does not encompass the heavy static model architectures. Instead, $D_{off}^{n}$ strictly represents the task-specific dynamic configurations (e.g., dynamic weights or mission-specific context parameters) required to initialize the remote server execution. Furthermore, the size of $D_{off}^{n}$ is fundamentally determined by the intrinsic structural characteristics of the specific DNN task and the chosen optimal DNN partition point (split point). Thus, one has

(7) $$\begin{array}{c} \sum_{m=1}^{M_{n}}r_{un}\left[m\right]\cdot t_{s}\geq \alpha_{un}\cdot D_{off} \end{array}$$

where $M_{n}=\left\lceil \frac{T_{n}^{lc}}{t_{s}}\right\rceil$ is the number of UAV line segments, in which the associated *u*-th UAV has to finish receiving the uploaded data from GT.

After phase two, the process moves to phase three, i.e., the *n*-th GT offloading the intermediate results $S_{n}$ to its associated UAV in a timely manner. Then, on receiving the offloaded data $D_{off}^{n}$ and intermediate result $S_{n}$, the MEC server on UAV *u* starts to execute the second part of the DNN program, which is phase four. Assuming the allocated processing speed by the UAV to *n*-th GT is $f_{un}^{oc}$, the execution time $T_{un}^{oc}$ on UAV can be defined as

(8) $$\begin{array}{c} T_{un}^{oc}=\frac{F_{n}\cdot D_{off}^{n}}{f_{un}^{oc}} \end{array}$$

We define $E_{nu}$ as the energy consumption of the UAV on computing task $U_{n}$, which can be formulated as

(9) $$\begin{array}{c} E_{un}=\varphi {\left(\alpha_{un}\cdot f_{un}^{oc}\right)}^{\vartheta } \end{array}$$

where φ is the effective switched capacitance, and ϑ≥1 is the positive constant [3,25]. In addition, during the whole UAV mission completion time, one UAV *u* only has limited computation capacity $F_{\max}^{u}$, which can be denoted as

(10) $$\begin{array}{c} \sum_{n=1}^{N}\alpha_{un}\cdot f_{un}^{oc}\leq F_{\max}^{u} \end{array}$$

Additionally, consider the whole task latency; the constraint for *n*-th GT uploading the intermediate result $S_{n}$ to the *u*-th UAV should be denoted as

(11) $$\begin{array}{c} \sum_{m=M_{n}+1}^{M_{n}^{\prime }}r_{un}\left[m\right]\cdot t_{s}\geq \alpha_{un}\cdot S_{n} \end{array}$$

where $M_{n}^{\prime }=\left\lceil \frac{T_{n}-T_{n}^{lc}-T_{un}^{oc}}{t_{s}}\right\rceil$, and $T_{n}-T_{n}^{lc}-T_{un}^{oc}$ is the time allowed for *n*-th GT to upload the intermediate result $S_{n}$ to the associated *u*-th UAV.

After the four phases following the PPO scheme, the associated UAV will transfer the final result to the GT via downlink, as shown in Figure 2. Because the result data is trivial and the downlink from UAV to GT normally has a high data transfer rate, we ignore the latency caused by the result downlink. To this end, through such a PPO scheme, each GT will have its deep learning task properly handled, with QoS on latency, by itself and together with one associate UAV in the air.

### 3.2. Problem Formulation

Let $Q=\{q_{u}\left[m\right],m\in M,u\in U\}$, $A=\{\alpha_{un},u\in U,n\in N\}$, and $F=\{f_{un}^{oc},u\in U,n\in N\}$, the optimization problem can be modeled as

(12) $$\begin{array}{c} P:\min_{Q,\;A,\;F}\;\max_{\forall u}\left(\sum_{m=1}^{M}E_{u}^{r-\mathrm{uav}}\left[m\right]+\beta \sum_{n=1}^{N}E_{un}\right) \end{array}$$

(12a) $$s.t.\;\;\alpha_{un}=\left\{0,1\right\},\;\sum_{u=1}^{U}\alpha_{un}\leq 1\;\forall u,n;$$

(12b) $$\sum_{n=1}^{N}\alpha_{un}\cdot f_{un}^{oc}\leq F_{\max}^{u},\;\;\;\forall u;$$

(12c) $$\sum_{m=1}^{M_{n}}r_{un}\left[m\right]\cdot t_{s}\geq \alpha_{un}\cdot D_{off},\;\;\;\forall u,n;$$

(12d) $$\sum_{m=M_{n}+1}^{M_{n}^{\prime }}r_{un}\left[m\right]\cdot t_{s}\geq \alpha_{un}\cdot S_{n},\;\;\;\forall u,n;$$

(12e) $$q_{u}\left[1\right]=q_{u}\left[M+1\right],\;\;\;\forall u;$$

(12f) $$∥q_{u}\left[m+1\right]-q_{u}\left[m\right]∥\leq \min\left\{t_{s}V_{\max}^{h},\Delta_{\max}^{h}\right\},\;\forall m,u;$$

where P is to minimize the maximum energy consumption of the UAVs while satisfying the QoS requirement of GT tasks on latency. To do so, we need to jointly optimize the UAV trajectory Q, GT-UAV association A, and the computing resource allocation F. Here, β>0 is the weighting parameter that balances the propulsion energy and the service (computation) energy; (12b) constrains the total computing capacity of each UAV; (12c) and (12d) enforce the QoS requirement that each task must be completed within its deadline; and (12e)∼(12f) constrain the UAV trajectory in the horizontal dimension. The problem P is non-convex due to the coupling between trajectory variables (in $r_{un}\left[m\right]$) and the binary association variables and thus cannot be solved directly in its current form.

### 3.3. Discussion on Model Assumptions

Before proceeding to the solution, we discuss several modeling assumptions and their justifications:

**Orthogonal sub-channel allocation.** We assume that orthogonal sub-channels are pre-allocated to avoid co-channel interference among UAV–GT links (cf. Equation (4)). While this simplifies the analysis, we acknowledge that in dense multi-UAV deployments, spectral scarcity and dynamic interference become significant concerns. Incorporating dynamic sub-channel allocation would scale the bandwidth parameter *B* by a fractional coefficient and introduce additional integer programming variables. We leave this extension to future work, noting that our current framework can accommodate it by treating the sub-channel allocation as an additional block in the BCD iteration.

**Neglecting GT antenna heights.** The LoS probability model in Equation (2) neglects the antenna heights of GTs. This is justified when the UAV altitude *H* (200 m in our simulations) is significantly larger than typical GT antenna heights (1–3 m), making the elevation angle approximation $\arctan\left(H/d_{un}\left[m\right]\right)\approx \arctan\left(H/d_{un}\left[m\right]+h_{GT}\right)$ highly accurate. In scenarios with substantial GT elevation differences (e.g., multi-story buildings), the model can be extended by replacing *H* with $H-h_{n}$ for each GT *n*.

**Constant velocity within segments.** The model assumes approximately constant horizontal velocity within each trajectory segment, which precludes explicit hovering (zero velocity). However, the rotary-wing propulsion model in Equation (1) naturally accounts for hovering energy (the $P_{0}$ and $P_{1}$ terms dominate at $v_{u}\left[m\right]\to 0$). In practice, the optimized trajectory may produce very short segments (small $∥q_{u}\left[m+1\right]-q_{u}\left[m\right]∥$) that approximate hovering behavior. For scenarios with very high terminal density where sustained hovering is optimal, the constraint (21a) can be relaxed by setting θ close to 1.

**UAV computation capacity.** The constraint $F_{\max}^{u}$ in Equation (10) represents the maximum sustainable computing frequency of the UAV’s onboard processor. This limit implicitly accounts for thermal dissipation constraints, as the maximum frequency is determined by the processor’s thermal design power (TDP) under the UAV’s cooling conditions. We do not model transient thermal dynamics, which is reasonable for the mission durations considered (minutes to tens of minutes).

## 4. Proposed Solution

To solve problem P, we employ a block coordinate descent algorithm to find the sub-optimal solution in an iterative way. In one iteration, we split P into three sub-problems. After a number of iterations, a suboptimal solution for the target problem can be achieved, while the block coordinate descent process reaches a predefined accuracy.

### 4.1. Optimize GT-UAV Association

Assuming UAV trajectory Q and the computational resource allocation F are pre-obtained and fixed, the optimization problem P can be simplified as

(13) $$\begin{array}{c} P1:\min_{A}\;\max_{\forall u}\left(\sum_{n=1}^{N}E_{un}\right) \end{array}$$

s.t.(12a)(12b)

(13a) $$\alpha_{un}\leq \frac{\sum_{m=1}^{M_{n}}r_{un}\left[m\right]\cdot t_{s}}{D_{off}},\;\;\;\forall u,n;$$

(13b) $$\alpha_{un}\leq \frac{\sum_{m=M_{n}+1}^{M_{n}^{\prime }}r_{un}\left[m\right]\cdot t_{s}}{S_{n}},\;\;\;\forall u,n;$$

which is to optimize the GT-UAV association. In P1, $r_{un}\left[m\right]$, $D_{off}$, $S_{n}$, $t_{s}$, $f_{un}^{oc}$ are constants, and (12c) and (12d) can be converted to be (13a) and (13b). To obtain the binary $\alpha_{un}=\left\{0,1\right\}$ in conjunction with other constraints, we relax the discrete variable to a continuous variable, i.e., $\alpha_{un}=\left[0,1\right]$. Then, as a continuous variable, $\alpha_{un}$ represents the likelihood of each GT associating to each individual UAV. To ensure that each GT can select and connect to only one optimal UAV, we take the uplink energy consumption of both GT and UAV together as the criterion to select the optimal UAV out of others in a finer grade. The uplink energy consumption of GT is defined as

(14) $$\begin{array}{cc} \; & E_{n}^{m}=P\cdot T_{n}^{lc},\;\;\;\forall n \end{array}$$

where *P* is the transmit power allocated by each GT; $T_{n}^{lc}=F_{n}\left(D_{n}-D_{off}^{n}\right)/f_{n}^{lc}$ is the data transferring time on uplink, which is the same time as the one GT spent on executing the local part of the deep learning task. To this end, problem $P_{1}$ can be further redefined as

(15) $$\begin{array}{cc} \; & P_{2}:\min_{A}\max_{\forall u}\left(\sum_{n=1}^{N}\left(E_{un}+\gamma \sum_{m=1}^{M_{n}}E_{n}\left[m\right]\right)\right)\\ \; & s.t.\;\;\;\alpha_{un}=\left\{0,1\right\},\;\left(12b)(13a)(13b\right) \end{array}$$

which can be directly solved by cvx, and obtain sub-optimal $\alpha_{un}$ as a series of continuous variables. Then, we convert $\alpha_{un}$ back into discrete variables as the GT-UAV association result,

(16) $$\begin{array}{cc} \; & \alpha_{un}=\left\lfloor \frac{\alpha_{un}}{\max_{\forall u}\left(\alpha_{un}\right)}\right\rfloor \end{array}$$

### 4.2. Optimize the Computational Resource Allocation of UAV

Assume the UAV trajectory Q and UAV–GT association A are fixed; the objective problem on F can be simplified as

(17) $$\begin{array}{cc} \; & P_{3}:\min_{F}\max_{\forall u}\left(\sum_{n=1}^{N}E_{un}\right)\\ \; & s.t.\;\;\;\sum_{n=1}^{N}\alpha_{un}\cdot f_{un}^{oc}\leq F_{\max}^{u},\;\;\;\forall u \end{array}$$

which is to optimize the computational resource allocation of UAV. In this problem, the execution time $T_{un}^{oc}$ is determined by the amount of computing resources allocated. Therefore, based on (8), the allocation of $f_{un}^{oc}$ follows the constraint

(18) $$\begin{array}{c} f_{un}^{oc}\geq \frac{F_{n}\cdot D_{off}^{n}}{T^{n}-T_{lc}^{n}} \end{array}$$

As a result, problem $P_{3}$ can be redefined as

(19) $$\begin{array}{cc} \; & P_{4}:\min_{F}\max_{\forall u}\left(\sum_{n=1}^{N}E_{un}\right)\\ \; & s.t.\;\;\;\;\left(12b)(18\right) \end{array}$$

which can be directly solved using the CVX tool.

### 4.3. Optimize the UAV Trajectory

After obtaining A and F, the optimization process continues to find the UAV trajectories, so as to minimize the UAV propulsion energy. Specifically, the UAV trajectory problem can be simplified to

(20) $$P_{5}:\min_{Q}\;\max_{\forall u}\sum_{m=1}^{M}E_{u}^{r-\mathrm{uav}}\left[m\right]$$

s.t.(12c)∼(12f)

which is still non-convex and cannot be solved directly.

To release the non-convexity, we convert $P_{5}$ to the following form:

(21) $$P_{6}:\min_{Q}\max_{\forall u}\sum_{m=1}^{M}∥∥q_{u}\left[m+1\right]-q_{u}{\left[m\right]∥}^{2}-{\left(t_{s}v_{u}^{*}\right)}^{2}∥$$

s.t.(12c)∼(12e)

(21a) $$∥q_{u}\left[m+1\right]-q_{u}{\left[m\right]∥}^{2}\geq {\left(\left(1-\theta \right)t_{s}v_{u}^{*}\right)}^{2}$$

(21b) $$∥q_{u}\left[m+1\right]-q_{u}{\left[m\right]∥}^{2}\leq \min\left\{{\left(\left(1+\theta \right)t_{s}v_{u}^{*}\right)}^{2},{\left(\Delta_{\max}^{h}\right)}^{2}\right\}$$

where $v_{u}^{*}$ represents the optimal horizontal velocity that minimizes the propulsion power in the horizontal dimension. The value of $v_{u}^{*}$ can be referenced in []. For rotary-wing UAVs, determining $V_{\max}^{h}$ directly from $E_{u}^{r-\mathrm{uav}}\left[m\right]$ is challenging. Fortunately, $V_{\max}^{h}$ can be efficiently approximated through numerical methods. Assume θ serves as a relaxation parameter; then the velocity *v* can be varied to be within the range of $\left(1-\theta \right)v_{u}^{*}$ to $\left(1+\theta \right)v_{u}^{*}$. Further, to relax the non-convexity of (12c) and (12d) caused by $r_{un}\left[m\right]$, we transform $r_{un}\left[m\right]$ to

(22) $$\begin{array}{cc} \; & r_{un}\left[m\right]=B\log_{2}\left(1+10^{-\frac{Ap_{un}\left[m\right]}{10}}\times \frac{\kappa_{un}\left[m\right]}{H^{2}+{d_{un}\left[m\right]}^{2}}\right) \end{array}$$

where $\kappa_{un}\left[m\right]=\frac{P}{BN_{0}}10^{-\frac{C}{10}}$. Due to $p_{un}\left[m\right]\geq 0$, expression (22) can be represented as

(23) $$\begin{array}{cc} \; & r_{un}\left[m\right]\geq B\log_{2}\left(1+\frac{\kappa_{un}\left[m\right]}{H^{2}+{d_{un}\left[m\right]}^{2}}\right) \end{array}$$

and,

(24) $$\begin{array}{cc} \; & r_{un}\left[m\right]\geq B\log_{2}\left(1+\xi_{un}\left[m\right]f\left(v_{un}\left[m\right]\right)\right) \end{array}$$

where $\xi_{un}\left[m\right]=\frac{\kappa_{un}\left[m\right]}{H^{2}}$,$f\left(x\right)=\frac{1}{1+x}$ and $v_{un}\left[m\right]=\frac{{d_{un}\left[m\right]}^{2}}{H^{2}}$.

It is evident that the formula concerning $v_{un}\left[m\right]$ above is a non-linear function. To make the problem more trackable, a taylor expansion of $v_{un}\left[m\right]$ is used with the local point $v_{un}^{l}\left[m\right]=\frac{{\left(d_{un}^{l}\left[m\right]\right)}^{2}}{H^{2}}$. Specifically, by substituting the local point into (24), we obtain the following expression by Taylor expansion:

(25) $$\begin{array}{cc} \; & \log_{2}\left(1+\xi_{un}\left[m\right]f\left(v_{un}\left[m\right]\right)\right)\geq \\ \; & \;\;\;\;J_{un}^{l}\left[m\right]\left(v_{un}\left[m\right]-v_{un}^{l}\left[m\right]\right)+W_{un}^{l}\left[m\right] \end{array}$$

where

(25a) $$\begin{array}{ccc} \; & J_{un}^{l}\left[m\right] & =\frac{-\xi_{un}\left[m\right]}{\ln2\left({\left(1+v_{un}^{l}\left[m\right]\right)}^{2}+\xi_{un}\left[m\right]\left(1+v_{un}^{l}\left[m\right]\right)\right)}, \end{array}$$

(25b) $$\begin{array}{ccc} \; & W_{un}^{l}\left[m\right] & =\log_{2}\left(1+\xi_{un}\left[m\right]f\left(v_{un}^{l}\left[m\right]\right)\right) \end{array}$$

Then, (12c) and (12d) can be transformed into the following forms:

(26) $$\begin{array}{cc} \; & \sum_{m=1}^{M_{n}}r_{un}\left[m\right]\geq B\sum_{m=1}^{M_{n}}(J_{un}^{l}\left[m\right]\left(v_{un}\left[m\right]-v_{un}^{l}\left[m\right]\right)\\ \; & \;\;\;\;+W_{un}^{l}\left[m\right])\geq \frac{\alpha_{un}\cdot D_{off}}{t_{s}} \end{array}$$

(27) $$\begin{array}{cc} \; & \sum_{m=M_{n}+1}^{M_{n}^{\prime }}r_{un}\left[m\right]\geq B\sum_{m=M_{n}+1}^{M_{n}^{\prime }}(J_{un}^{l}\left[m\right]\left(v_{un}\left[m\right]-v_{un}^{l}\left[m\right]\right)\\ \; & \;\;\;\;+W_{un}^{l}\left[m\right])\geq \frac{\alpha_{un}\cdot S_{n}}{t_{s}} \end{array}$$

In this way, the objective problem (21) can be simplified into a convex Quadratic Constraint Quadratic Programming (QCQP) problem. The new objective problem is:

(28) $$\begin{array}{cc} \; & P_{6}:\min_{Q}\max_{\forall u}\sum_{m=1}^{M}∥∥q_{u}\left[m+1\right]-q_{u}{\left[m\right]∥}^{2}-{\left(t_{s}v_{u}^{*}\right)}^{2}∥\\ \; & s.t.\;\left(12e)(21a)(21b)(26)(27\right) \end{array}$$

which can be directly solved using CVX.

### 4.4. Overall Algorithm Design

Based on previous works, the overall block coordinate descent algorithm can be designed as Algorithm 1. In this algorithm, we set the UAV’s initial trajectory to be an elliptical trajectory and the UAV altitude to be fixed. The elliptical initialization is chosen for three practical reasons: (i) *operational convention*—in the absence of strong target guidance, UAVs naturally adopt circular or elliptical loitering patterns (similar to aircraft holding patterns at airports), which represent the most energy-efficient cruise trajectories by avoiding sharp turns and abrupt accelerations; (ii) *fair coverage*—an elliptical path provides the most uniform initial coverage probability across all ground terminals, ensuring no GT is systematically disadvantaged at initialization; and (iii) *constraint satisfaction*—elliptical trajectories inherently satisfy the return constraint $q_{u}\left[1\right]=q_{u}\left[M+1\right]$ and velocity limits while remaining smooth and differentiable, guaranteeing feasibility of the initial solution for the subsequent convex optimization steps. We differentiate the initial flight paths of each UAV by assigning different ellipse centers and orientations based on the spatial distribution of GTs.
**Algorithm 1:** SCA-BCD Joint Optimization Algorithm
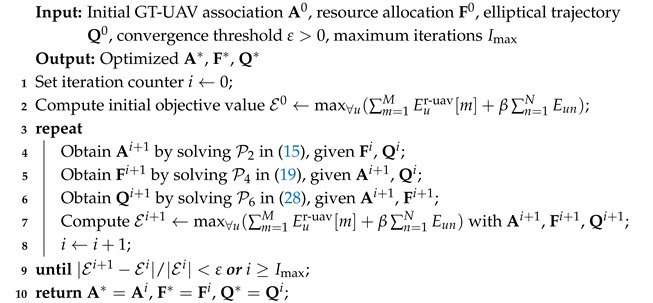


The algorithm terminates when either the relative change in the objective value falls below the threshold ε (set to $10^{-3}$ in our simulations), or the maximum number of iterations $I_{\max}$ (set to 100) is reached. Since each subproblem is solved optimally (or to a guaranteed approximation via SCA), the objective value $E^{i}$ is monotonically non-increasing across iterations. Combined with the lower bound of zero on energy consumption, convergence is guaranteed.

**Illustrative Example.** Consider a simplified scenario with U=2 UAVs and N=3 GTs. In iteration i=0, both UAVs start with elliptical trajectories. Step 3 solves the association problem—suppose GT 1 and GT 2 are assigned to UAV 1, and GT 3 to UAV 2. Step 4 allocates computing resources proportionally to each UAV’s assigned workload. Step 5 optimizes trajectories so that UAV 1 moves closer to GT 1 and GT 2, while UAV 2 adjusts toward GT 3. In iteration i=1, with the new trajectories, the association may be refined (e.g., GT 2 might switch to UAV 2 if UAV 2’s new position offers better link quality), resources are reallocated accordingly, and trajectories are further refined. This process repeats until convergence, typically within 30–50 iterations.

The time complexity of Algorithm 1 (the block coordinate descent algorithm) is determined by the convergence iteration count and the complexity of solving its three subproblems per iteration. Let *U* denote the number of UAVs, *N* the number of ground terminals (GTs), *M* the number of discrete trajectory segments per UAV, and *K* the number of iterations required for convergence. In each iteration, optimizing GT-UAV associations and UAV computational resource allocation both form convex programs with a complexity of $O({\left(U\cdot N\right)}^{3})$. Optimizing UAV trajectories, transformed via successive convex approximation (SCA) into a QCQP yields a complexity of $O({\left(U\cdot M\right)}^{3})$. Thus, the per-iteration worst-case complexity is $O({\left(U\cdot N\right)}^{3}+{\left(U\cdot M\right)}^{3})$.

Crucially, while this polynomial complexity exists per iteration, the algorithm strictly converges within a small constant number of iterations (typically K≈50). In our simulations on a standard PC, the average wall-clock time required for overall convergence is merely 3 to 5 s. In stark contrast, Deep Reinforcement Learning (DRL) algorithms widely used in similar domains, such as Deep Deterministic Policy Gradient (DDPG), suffer from severe training bottlenecks. DDPG requires continuous interactions with the environment over tens of thousands of episodes to optimize its actor-critic networks. Even with offline training, adapting DDPG to dynamically changing GT tasks inherently takes hours of wall-clock time. Therefore, our proposed SCA-based framework guarantees a highly computationally efficient and real-time responsive solution suitable for time-sensitive MEC-enabled UAV missions.

## 5. Simulation

In this section, the proposed optimization algorithm is validated through simulations using the CVX tool in MATLAB. The simulation considers a 500 m × 500 m area with a moderate density of GTs to demonstrate the practicality of the approach. Three UAVs are deployed from the same starting point, and the system parameters are listed in Table 3.

**Parameter Justification.** The simulation parameters are chosen based on established references and practical considerations. The path loss parameters (a=9.61, b=0.16, $\eta_{\mathrm{LoS}}=1$ dB, $\eta_{\mathrm{NLoS}}=20$ dB) correspond to a suburban environment as specified in the ITU-R urban/suburban channel model widely adopted in UAV communication literature [3,28]. The rotary-wing UAV propulsion parameters ($U_{\mathrm{tip}}$, $v_{0}$, $d_{0}$, *s*, ρ, *G*) follow the validated model in [5], which is calibrated against real flight data of commercial multi-rotor platforms. The UAV altitude of 200 m balances LoS probability (higher altitude improves LoS) against path loss (higher altitude increases distance). The computation energy parameters ($\varphi =10^{-9}$, ϑ=3) are standard values for CMOS-based processors as established in the MEC literature [29]. The GT task parameters (data size 1–5 Mb, deadline 60–70 s) represent typical lightweight DNN inference tasks such as object detection models (e.g., YOLOv5-small).

**Performance Metrics.** We evaluate the following metrics to comprehensively assess the proposed algorithm: (i) *UAV propulsion energy consumption*—quantifying the flight energy cost, which is the dominant energy component and directly reflects trajectory efficiency; (ii) *service energy consumption*—measuring the computational energy spent on processing offloaded tasks, reflecting resource allocation efficiency; (iii) *computing resource allocation (CDF)*—showing the distribution of allocated CPU cycles across UAVs, indicating workload balance; and (iv) *uplink data transmission (CDF)*—characterizing the communication performance achieved under different trajectory schemes.

**Figure 3:**
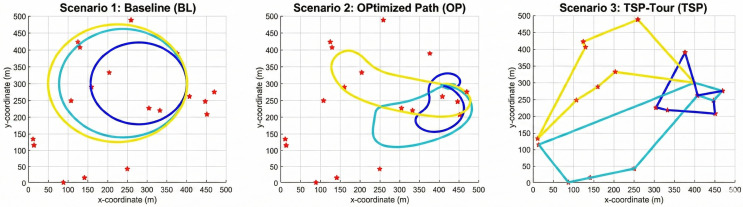
The trajectories of three UAVs under various scenarios.

**Figure 4:**
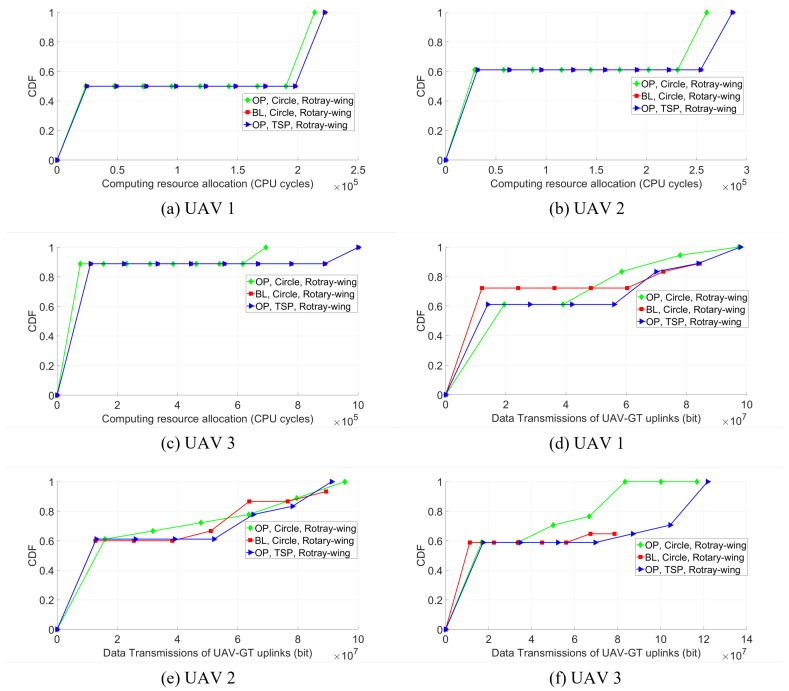
Allocation of computing resources (**a**–**c**) and data transmissions (**d**–**f**) of GT tasks for UAV1, UAV2, and UAV3, respectively.

**Figure 5:**
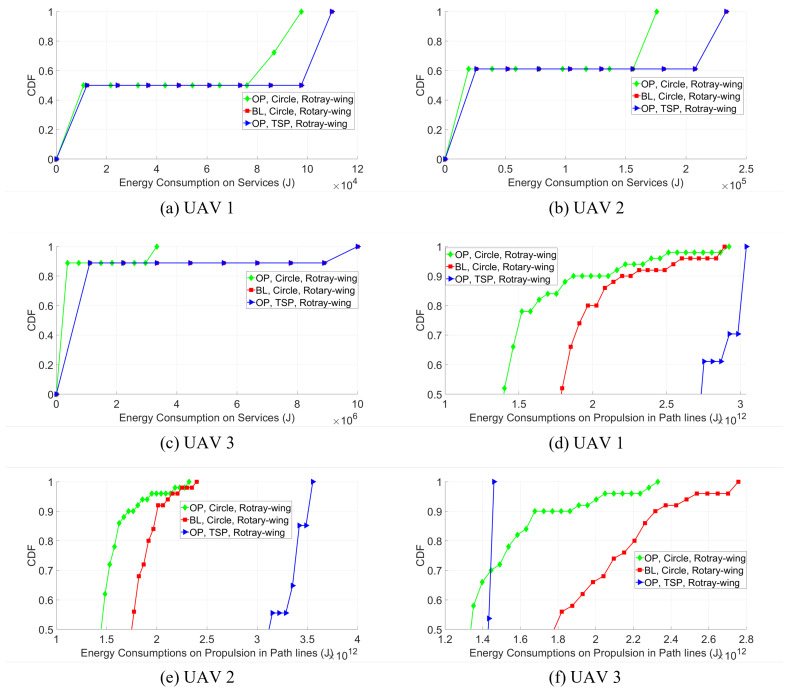
The service energy (**a**–**c**) and propulsion energy (**d**–**f**) consumption of UAV1, UAV2, and UAV3, respectively, under different trajectories.

We compare the optimized solution (OP) with the baseline solution (BL) in terms of both quality of service and energy consumption. In the BL setting, the UAV trajectory is adopted as the initial elliptical trajectory of Algorithm 1. For the GT-UAV associations, the strategy in BL is that each UAV selects the GTs with the best link quality based on proximity. Meanwhile, we compare the optimized solution to the Traveling Salesman Problem (TSP) solution, where each UAV flies to the closest position to each of its associated GTs, providing the highest service quality at the cost of increased propulsion energy. The TSP trajectories are determined using the GT-UAV associations established following the same method as in the BL approach, but the TSP method lacks iterative refinement capability. Based on these settings, we present three UAV trajectories in Figure 3, followed by the service and propulsion energy consumption comparisons in Figure 4 and Figure 5.

Figure 3 illustrates the trajectories of each UAV under different schemes. Scenario 1 represents the results of a BL solution, where the trajectories of the three UAVs are not identical but all take an elliptical shape, covering a slightly larger range for comparison purposes. Scenario 2 shows the UAV trajectories under the TSP solution. After selecting their associated GTs, each UAV finds the shortest path through these GTs using the TSP solution, starting from the same departure point. The advantage of TSP trajectories lies in minimizing the distance between the UAV and GT. Scenario 3 presents the UAV trajectories led by OP. It is evident that the UAV flight paths are neither too close to the GTs nor too far from them. This optimization approach effectively balances service quality and the energy consumption on UAV propulsion energy and service providing.

Figure 4 presents the CDF of computing resource allocation in subfigures (a–c) and the CDF of uplink data transmission rates in subfigures (d–f), where each column corresponds to UAV1, UAV2, and UAV3, respectively. As shown in subfigures (a–c), the optimized UAV computing resource allocation is significantly lower than that of TSP and BL schemes. TSP and BL schemes adopt the same set of GT-UAV associations, which are randomly generated and not iteratively optimized. After the GT-UAV association is determined, the total computing resources are evenly allocated directly. Therefore, the images of TSP and BL in Figure 4a–c are the same. However, the OP iteratively optimizes both the GT-UAV association and computing resource allocation so that the task is completed with minimal computing resource consumption.

Figure 5 shows the service energy consumption in subfigures (a–c) and the propulsion energy consumption in subfigures (d–f), where each column corresponds to UAV1, UAV2, and UAV3, respectively. As shown in subfigures (a–c), under the OP solution, the service energy consumption differs from that of the TSP and BL solutions. Both TSP and BL solutions do not iteratively optimize the GT-UAV associations; they merely make a single selection based on initial trajectories. Moreover, the task load for each GT may vary. In contrast, the OP simultaneously optimize both the GT-UAV associations and the trajectories in an iterative manner, resulting in each UAV in the OP scenario choosing a path that minimizes the energy consumption.

However, under the TSP method, while the UAV’s service energy consumption is lower than that under BL, its propulsion energy consumption is considerably higher. Firstly, the initial trajectories in the BL solution are not optimized and are simply elliptical, leading to relatively higher propulsion energy consumption. This can be observed from Figure 5d–f, where the optimized trajectories clearly result in lower propulsion energy consumption. However, TSP trajectories tend to be closer to the GTs compared to elliptical trajectories. This means that when linking to a larger number of GTs, the UAV has to fly longer distances, leading to higher energy consumption. However, when the number of GTs to be linked is small and their positions are relatively concentrated, TSP’s energy consumption can be the lowest. For example, in Figure 5f, the UAV’s energy consumption under the TSP approach is somewhat lower compared to OP and BL, due to the more centralized GT placement. In summary, the optimization solution we proposed is superior to the baseline approach and meets the energy-saving requirements of UAV-enabled Mobile Edge Networks.

**Figure 6:**
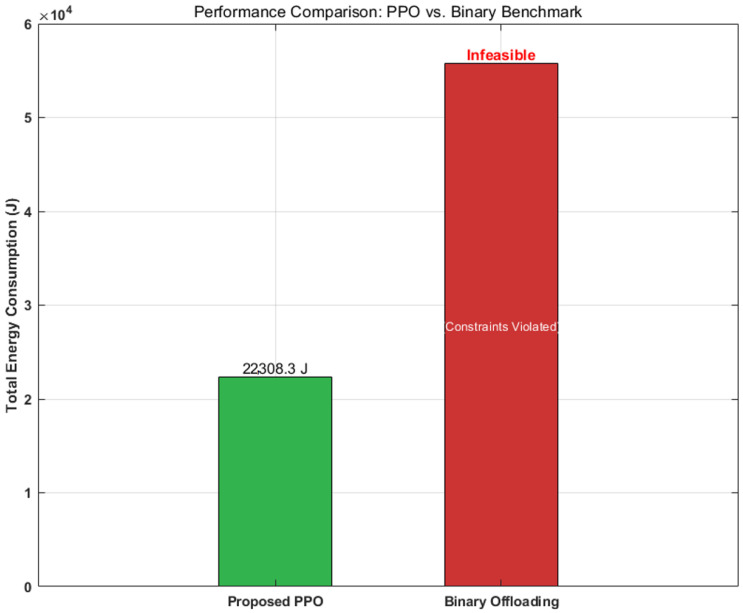
Performance Comparison: Proposed PPO vs. Binary Offloading Benchmark.

To further validate the necessity and superiority of the proposed Partial Program Offloading (PPO) scheme, we introduce an additional simulation comparing it against a benchmark “Binary Offloading” scheme. In the Binary Offloading scheme, deep learning tasks cannot be partitioned and must be fully offloaded to the remote UAVs (i.e., $D_{off}^{n}=D_{n}$). As shown in Figure 6, under the identical stringent task latency deadlines and UAV computing capacity constraints, the Binary Offloading scheme fails to find a feasible solution (**Infeasible**). The massive volume of transmission data and intensive computation strictly requested by full offloading severely overloads the limited bandwidth and UAV capabilities. In stark contrast, our proposed PPO scheme effectively obtains an optimal solution with a total energy consumption of approximately $2.23\times 10^{4}$ J. By dynamically adjusting the offloading ratio based on channel qualities and task requirements, the PPO scheme intelligently balances the local and remote computing burdens, proving its absolute necessity in resource-constrained UAV-MEC systems.

**Figure 7:**
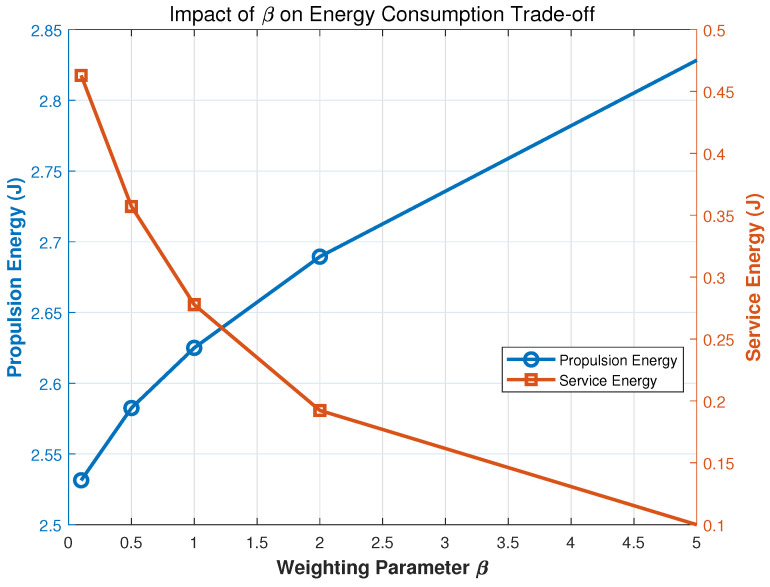
The trade-off curve between UAV propulsion energy and service energy consumption under varying weighting parameter β.

Furthermore, to investigate the impact of the weighting parameter β (defined in the objective function Equation (12)) on the system performance, we conducted a sensitivity analysis experiment. β serves as a critical lever to balance the UAV propulsion energy and the service energy consumption. As illustrated in Figure 7, a distinct trade-off curve is observed. When β is relatively small, the optimization algorithm prioritizes minimizing the UAV propulsion energy, resulting in energy-efficient flight trajectories but higher service energy costs. Conversely, as β increases, the system places a heavier penalty on service energy. Consequently, the UAVs adapt their trajectories—often flying closer to the GTs to secure better channel qualities—and optimize resource allocation to drastically reduce the service energy consumption. This reduction comes at the cost of a slight increase in propulsion energy due to the extra maneuvering. This trade-off validates that our proposed joint optimization scheme can flexibly adapt to diverse mission priorities (e.g., prioritizing communication quality vs. extending UAV flight endurance) by simply tuning the parameter β.

Finally, we compared the uplink data transfer rates of GT and UAV. It can be seen from Figure 4d–f that TSP has the highest speed among the three modes, while OP is very close to BL. Because our main goal is to reduce energy consumption, the existing bit rate is in line with our expectations.

## 6. Conclusions

In this paper, we investigated the joint optimization of UAV trajectories, computing resource allocation, and partial deep learning task offloading in MEC-enabled multi-UAV systems. We designed a Partial Program Offloading (PPO) scheme that splits DNN tasks at an optimal layer to balance local and remote computation and formulated a min-max energy optimization problem subject to task latency and UAV mobility constraints. To solve this non-convex coupled problem, we proposed an SCA-BCD algorithm that decomposes it into three tractable convex subproblems solved iteratively. Simulation results demonstrated that the proposed approach significantly reduces UAV energy consumption compared to baseline and TSP-based solutions while satisfying all QoS requirements. We further validated the necessity of partial offloading by showing that binary offloading becomes infeasible under identical resource constraints.

**Discussion on Security and Trust.** While this work focuses on optimizing energy efficiency and QoS, the practical deployment of MEC-enabled multi-UAV systems also requires addressing security concerns. In particular, when GTs offload sensitive deep learning tasks (e.g., surveillance data processing) to UAVs, ensuring that all participating UAVs are properly authenticated and trustworthy is critical. Malicious or compromised UAVs could intercept offloaded data, inject false computation results, or disrupt the cooperative optimization process. Recent works have proposed cross-layer physical-layer authentication techniques for UAV communications [30], which can complement our optimization framework by providing lightweight UAV identity verification without additional cryptographic overhead. Integrating trust-aware offloading decisions—where GTs preferentially offload to authenticated UAVs—into our BCD framework represents a promising direction for future work.

**Future Work.** We plan to advance this work in several directions: (i) expanding the experimental scale to evaluate scalability with larger numbers of UAVs and GTs, and conducting a systematic study of how increasing GT density affects the convergence speed of the BCD algorithm—specifically, characterizing the relationship between *N* and the number of iterations required to reach a prescribed accuracy ε; (ii) investigating the trade-off between UAV computation speed (i.e., the allocated CPU frequency $f_{un}^{oc}$) and total mission energy consumption, with the goal of deriving a Pareto-optimal frontier that guides the selection of the weighting parameter β; (iii) incorporating dynamic sub-channel allocation to address spectral scarcity in dense deployments; (iv) extending the model to support multimodal deep learning tasks (e.g., joint image and speech processing) with heterogeneous split-point selection; (v) incorporating physical heat dissipation limits and thermodynamic constraints of onboard hardware into the resource allocation model; and (vi) investigating hybrid approaches that combine our SCA-BCD framework with Deep Reinforcement Learning for scenarios requiring online adaptation to rapidly changing environments.

## Figures and Tables

**Table 1:** Comparison with Related Works.

Ref.	UAV Config.	Opt. Dimensions	Task Type	Method
[6,7,8]	Single UAV	Trajectory only	General computation	SCA
[27]	Multi-UAV	Trajectory + power	Data transmission	SCA + BCD
[9,10]	Multi-UAV	Offloading only	Deep learning (full)	DRL
[13]	Multi-UAV	Trajectory + energy + placement	General (full)	Triple-RL
[18]	Multi-UAV	Clustering + scheduling	General (full)	RL + Game
[18]	Multi-UAV	Trajectory + resource	Communication	Heuristic
This Work	Multi-UAV	Trajectory + resource + partial offloading	Deep learning (partial)	SCA + BCD

**Table 2:** Summary of Key Notations.

Symbol	Description
*U*, *N*, *M*	Number of UAVs, GTs, and discrete trajectory segments
U, N, M	Index sets of UAVs, GTs, and trajectory segments
$q_{u}\left[m\right]$	Horizontal coordinate $\left(x_{u}\left[m\right],y_{u}\left[m\right]\right)$ of the *u*-th UAV at the *m*-th waypoint
*H*	Fixed flight altitude of all UAVs
$w_{n}$	Horizontal location $\left(x_{n},y_{n}\right)$ of the *n*-th GT
$\alpha_{un}$	Binary association variable: $\alpha_{un}=1$ if GT *n* offloads to UAV *u*, 0 otherwise
$f_{un}^{oc}$	Computing resource (CPU frequency) allocated by UAV *u* to GT *n*
$D_{n}$, $D_{off}^{n}$	Total task data size and offloaded portion for GT *n*
$F_{n}$	Required computing intensity (CPU cycles per bit) for GT *n*’s task
$T_{n}$	Task completion deadline for GT *n*
$R_{n}$	Ratio of intermediate result size to input data size
$S_{n}=D_{n}\times R_{n}$	Intermediate result data size
$r_{un}\left[m\right]$	Achievable uplink rate from GT *n* to UAV *u* in segment *m*
$t_{s}$	Fixed time duration per trajectory segment
$\Delta_{\max}^{h}$	Maximum horizontal displacement per segment
$V_{\max}^{h}$	Maximum horizontal velocity of UAVs
$v_{u}\left[m\right]$	Horizontal velocity of UAV *u* in segment *m*
β	Weighting parameter balancing propulsion and service energy
$F_{\max}^{u}$	Maximum computing capacity of UAV *u*

**Table 3:** Parameter settings of the simulation.

Parameter	Value
Bandwidth *B*, DL power: $P_{\max}$	2 GHz, 5 mW
Pathloss: *a*, *b*, $\eta_{\mathrm{Los}}$, $\eta_{\mathrm{NLos}}$	9.61, 0.16, 1, 20
Rotary-wing: $U_{\mathrm{tip}}$,$v_{0}$,$d_{0}$	120, 4.3, 0.6
Rotary-wing: *s*, ρ, *G*	0.05, 1.225, 0.503
$P_{0}$, $P_{1}$	$\frac{12*30^{3}*0.4^{3}}{8}\rho sG$, $\frac{1.1*20^{3/2}}{\sqrt{2\rho G}}$
Rotary-wing: $V_{\max}^{h}$, $V_{\max}^{v}$	10 m/s, 10 m/s
Noisy density: $N_{0}$	−169 dBm/Hz
GT task: $T_{k}$,$D_{k}$	60∼70 s, 1∼5 Mb
Energy on computation: φ,ϑ	$10^{-9}$, 3
Computing capacity: $f_{n}^{lc}$	$2\times 10^{5}∼3\times 10^{5}$ CPU cycles
GT task: $F_{k}$	$6\times 10^{6}∼8\times 10^{6}$ CPU cycles
UAV height: $h_{m}$	200 m
Number of GTs and UAVs: *N*,*U*	18, 3
Time episodes: *M*	50

## Data Availability

The data presented in this study are available on request from the corresponding author.

## Abbreviations

The following abbreviations are used in this manuscript:

UAV	Unmanned Aerial Vehicle
GT	Ground Terminal
MEC	Mobile Edge Computing
SCA	Successive Convex Approximation
BCD	Block Coordinate Descent
DRL	Deep Reinforcement Learning
TSP	Traveling Salesman Problem
BL	Baseline
OP	Optimized Proposal
PPO	Partial Program Offloading
QoS	Quality of Service
DDPG	Deep Deterministic Policy Gradient
DDQN	Double Deep Q-Network
CDF	Cumulative Distribution Function
